# Sociodemographic factors associated with treatment-seeking and treatment receipt: cross-sectional analysis of UK Biobank participants with lifetime generalised anxiety or major depressive disorder

**DOI:** 10.1192/bjo.2021.1012

**Published:** 2021-11-19

**Authors:** Christopher Rayner, Jonathan R. I. Coleman, Kirstin L. Purves, Ewan Carr, Rosa Cheesman, Molly R. Davies, Jaime Delgadillo, Christopher Hübel, Georgina Krebs, Alicia J. Peel, Megan Skelton, Gerome Breen, Thalia C. Eley

**Affiliations:** Social, Genetic and Developmental Psychiatry Centre, Institute of Psychiatry, Psychology & Neuroscience, King's College London, UK; Social, Genetic and Developmental Psychiatry Centre, Institute of Psychiatry, Psychology & Neuroscience, King's College London, UK; and UK National Institute for Health Research Biomedical Research Centre, South London and Maudsley NHS Trust, UK; Social, Genetic and Developmental Psychiatry Centre, Institute of Psychiatry, Psychology & Neuroscience, King's College London, UK; Department of Biostatistics and Health Informatics, King's College London, UK; Social, Genetic and Developmental Psychiatry Centre, Institute of Psychiatry, Psychology & Neuroscience, King's College London, UK; Social, Genetic and Developmental Psychiatry Centre, Institute of Psychiatry, Psychology & Neuroscience, King's College London, UK; and UK National Institute for Health Research Biomedical Research Centre, South London and Maudsley NHS Trust, UK; Clinical Psychology Unit, Department of Psychology, University of Sheffield, UK; Social, Genetic and Developmental Psychiatry Centre, Institute of Psychiatry, Psychology & Neuroscience, King's College London, UK; UK National Institute for Health Research Biomedical Research Centre, South London and Maudsley NHS Trust, UK; and Department of Medical Epidemiology and Biostatistics, Karolinska Institutet, Sweden; Social, Genetic and Developmental Psychiatry Centre, Institute of Psychiatry, Psychology & Neuroscience, King's College London, UK; and National and Specialist OCD, BDD and Related Disorders Clinic for Young People, South London and Maudsley NHS Foundation Trust, UK; Social, Genetic and Developmental Psychiatry Centre, Institute of Psychiatry, Psychology & Neuroscience, King's College London, UK; Social, Genetic and Developmental Psychiatry Centre, Institute of Psychiatry, Psychology & Neuroscience, King's College London, UK; Social, Genetic and Developmental Psychiatry Centre, Institute of Psychiatry, Psychology & Neuroscience, King's College London, UK; and UK National Institute for Health Research Biomedical Research Centre, South London and Maudsley NHS Trust, UK; Social, Genetic and Developmental Psychiatry Centre, Institute of Psychiatry, Psychology & Neuroscience, King's College London, UK; and UK National Institute for Health Research Biomedical Research Centre, South London and Maudsley NHS Trust, UK

**Keywords:** Anxiety disorders, depressive disorders, primary care, patients, epidemiology

## Abstract

**Background:**

Anxiety and depressive disorders can be chronic and disabling. Although there are effective treatments, only a fraction of those impaired receive treatment. Predictors of treatment-seeking and treatment receipt could be informative for initiatives aiming to tackle the burden of untreated anxiety and depression.

**Aims:**

To investigate sociodemographic characteristics associated with treatment-seeking and treatment receipt.

**Method:**

Two binary retrospective reports of lifetime treatment-seeking (*n* = 44 810) and treatment receipt (*n* = 37 346) were regressed on sociodemographic factors (age, gender, UK ethnic minority background, educational attainment, household income, neighbourhood deprivation and social isolation) and alternative coping strategies (self-medication with alcohol/drugs and self-help) in UK Biobank participants with lifetime generalised anxiety or major depressive disorder. Analyses were also stratified by gender.

**Results:**

Treatment access was more likely in those who reported use of self-help strategies, with university-level education and those from less economically advantaged circumstances (household income <£30 000 and greater neighbourhood deprivation). Treatment access was less likely in those who were male, from a UK ethnic minority background and with high household incomes (>£100 000). Men who self-medicated and/or had a vocational qualification were also less likely to seek treatment.

**Conclusions:**

This work on retrospective reports of treatment-seeking and treatment receipt at any time of life replicates known associations with treatment-seeking and treatment receipt during time of treatment need. More work is required to understand whether improving rates of treatment-seeking improves prognostic outcomes for individuals with anxiety or depression.

In the UK, one in six adults experience clinically significant anxiety or depressive symptoms in a given week, but only one in three with symptoms are receiving treatment.^1^ The proportion of cases who meet diagnostic criteria for a mental disorder but who do not seek or receive treatment is called the treatment gap.^2^ This consists of the proportion of cases who do not seek treatment, defined here as the seeking gap, and the proportion of cases who seek treatment but do not eventually receive it called the access gap.^3^ Given the high prevalence and burden of anxiety and depressive disorders and the scale of the treatment gap, work is urgently required to identify barriers to treatment. Individuals might not seek treatment for a variety of reasons, including poor mental health literacy, lack of awareness about treatment options, negative beliefs about specific treatments and stigma.^4,5^ Understanding these factors will enable the development of materials to improve mental health literacy and attitudes toward treatments.^6^ It is also important to identify who public health strategies should target, and to think about how interventions can be tailored to different groups to encourage healthy treatment-seeking behaviours. Some evidence has been found for demographic associations with treatment receipt. In the UK, those who are White British, female or middle-aged are most likely to report having received treatment during a 12-month period.^1^ Existing studies have largely focused on demographic associations with treatment receipt during a discrete period, which is likely to be within the context of a single episode of poor mental health. Generalised anxiety disorder (GAD) and major depressive disorder (MDD) are often chronic, and can substantially reduce quality of life and productivity.^7,8^ Thus, enabling earlier treatment access has considerable potential for reducing the impact of anxiety and depression on educational and occupational outcomes. It will be necessary to improve rates of both treatment-seeking and treatment receipt to reduce the burden of untreated anxiety and depression. Strategies to improve treatment-seeking, and so reduce the seeking gap, will need to be aimed at the general population and targeted to specific groups (e.g. men).^9^ In contrast, strategies to improve treatment receipt and subsequent engagement, and so reduce the access gap, need to be actioned by health professionals, to ensure that screening, diagnosis and treatment selection accounts for social and cultural differences that exist in the population.^10^ This can be advanced by identifying at-risk groups, with whom further work can be done to investigate mediating influences as psychosocial barriers to treatment-seeking and treatment receipt.

Using retrospective self-report at a single time point, we tested for associations with lifetime (i.e. ever versus never) treatment-seeking and treatment receipt in adults who met diagnostic criteria for a lifetime GAD or MDD diagnosis. Our focus on both treatment-seeking and treatment receipt was inspired by evidence that individuals living in more socially deprived areas are more likely to have sought, but not actually received treatment.^11^ Finally, there is evidence to suggest that there are gender differences in treatment-seeking and treatment receipt, and in alternative coping mechanisms such as alcohol and drug use.^12^ To test whether sociodemographic factors vary in their association with treatment-seeking according to gender, we performed gender-stratified analyses. This information might also be important when developing targeted interventions for men and women. In sum, there is some evidence for factors associated with treatment receipt during a given episode of illness, but virtually none exploring factors associated with a lack of ever seeking and receiving treatment. This is particularly important as public health work to promote treatment-seeking to those at risk of not doing so could have considerable benefit both to individuals and society, by avoiding longer, more chronic illness.

## Method

### Participants

In total, 9 238 453 UK residents were invited to take part in the UK Biobank study, and 503 328 individuals aged 40–70 years were recruited at baseline (2006–2010).^13^ At baseline, data were collected on sociodemographic characteristics, medical histories and health and lifestyle-related factors. An online mental health questionnaire (MHQ) was completed by 157 366 participants at follow-up (2016–2017).^14^ Baseline participants were more likely to be older, female, identify as White British, have favourable health status and be from more economically advantaged neighbourhoods, compared with the general population.^15^ At MHQ follow-up, respondents were also more likely to be female, healthier and from more economically advantaged circumstances than UK Biobank participants who did not respond.^16^ Participants were considered for analysis if they completed the MHQ, endorsed one of four screening questions (*n* = 96 906) and met the full diagnostic criteria for GAD or MDD (*n* = 47 013). Analyses were restricted in this manner because we were interested in detecting factors associated with treatment-seeking and treatment receipt in those who were most likely to have clinically relevant treatment needs, so that findings would be generalisable to this specific group. Thus, 123 662 participants who completed the MHQ were ineligible and excluded from analyses. Participants were also excluded from analyses if they had discordant data on treatment-seeking (*n* = 1336), diagnoses (*n* = 883) and medication use (*n* = 393).

### Ethics and consent

UK Biobank has research ethics approval from the North West Multi-centre Research Ethics Committee (MREC; approval number 11/NW/0382) which covers the whole UK. It also sought the approval in England and Wales from the Patient Information Advisory Group (PIAG) for gaining access to information that would allow it to invite people to participate. PIAG has since been replaced by the National Information Governance Board for Health & Social Care (NIGB). In Scotland, UK Biobank has approval from the Community Health Index Advisory Group (CHIAG). Participation in the UK Biobank is voluntary, and participants are free to withdraw at any time. Informed written consent was obtained by participants at baseline. The current study was performed under UK Biobank application 18177. All relevant ethical guidelines have been followed during the analysis of this data.

### Measures

#### Symptoms of anxiety and depression: screening, diagnosis and controlling for symptom severity

The MHQ assessed common mental health problems, including lifetime symptoms of anxiety, depression and experiences of healthcare. Participants were initially asked whether they had experienced ‘worry for a period of 6 months or longer’, ‘worry more than most people would in a similar situation’, ‘prolonged loss of interest in normal activities’ or ‘prolonged feelings of sadness or depression’. Those who endorsed an anxiety or depression screening symptom question were subsequently asked about additional anxiety or depression symptoms, respectively, from which diagnoses were made with the Composite International Diagnostic Interview Short-Form (CIDI-SF^17^). Screened participants (those reporting at least one symptom) were also asked about their history of treatment-seeking and treatment receipt. Therefore, if participants did not report experience of at least one of four screening symptoms, they were not asked about additional symptoms, nor were they asked about their history of treatment. Of 157 366 MHQ respondents, 96 096 experienced at least one of four symptom screening items and 47 013 reported a sufficient number, severity and impact of symptoms to meet DSM-IV diagnostic criteria for a lifetime diagnosis of GAD (*n* = 11 110; prevalence = 7%) or MDD (*n* = 41 971; prevalence = 27%), as determined by the CIDI-SF. Prevalence rates estimated here are larger, but comparable to lifetime prevalence estimates reported in a population-representative sample for GAD (prevalence = 5%) and MDD (prevalence = 21%), respectively.^14^ The skip logic of CIDI-SF and self-report of symptoms are limitations of the study design. However, we aimed to generate a sample of individuals likely to have experienced clinical symptom burden to assess factors that might influence treatment-seeking. The CIDI-SF has a high classification accuracy for MDD and GAD.^17^ Further information on the validity and reliability of composite measures used by this questionnaire are detailed elsewhere.^14,17^

We expected that reporting of symptom severity and impairment was likely to be the strongest influence on treatment-seeking, as these reflect a greater recognition of a need for treatment. To estimate the effects of sociodemographic factors on treatment-seeking and treatment receipt holding symptom severity constant, we included a continuous measure of overall symptom severity as a covariate in multivariable models. Some participants only completed assessment for one or other diagnosis. For each set of symptoms (GAD and MDD), a symptom severity score was computed as the proportion of symptoms endorsed, weighted by the reported impact on roles and day-to-day functioning for each set of symptoms. If scores for both anxiety and depression were available, the larger of these scores was carried forward as the symptom severity score. Symptom scores were standardised (mean 0, s.d. 1).

#### Treatment-related outcomes

Treatment-seeking (yes/no) was derived from participants who, in response to the MHQ, reported on whether they had sought help from a professional in response to their symptoms of anxiety or depression. Treatment receipt (yes/no) was assessed in participants who reported ‘yes’ to treatment-seeking. Those who reported receiving prescribed medication or talking therapy were defined as treatment receivers, and those who received neither prescribed medication nor talking therapy after having sought treatment were the comparison group. Therefore, treatment receipt participants are the subset of participants that reported ‘yes’ to treatment-seeking (Supplementary Figure 1 available at https://doi.org/10.1192/bjo.2021.1012). In response to the same four symptoms, participants were asked ‘Did you ever use the following for [the symptom] or the problems it caused?’, with the options ‘(i) Drugs or alcohol’, ‘(ii) Unprescribed medication’ and ‘(iii) Other therapeutic activities [mindfulness, yoga, or art classes]’. To investigate whether use of alternative coping strategies impacts on treatment-seeking and treatment receipt, self-medication with alcohol/drugs (item i: yes/no) and self-help (items ii and iii combined: yes/no) were included as explanatory variables in our analysis.

#### Sociodemographic factors

Sociodemographic variables were collected at baseline. For our analysis, these included age, gender, ethnicity, educational attainment, household income, neighbourhood deprivation and social isolation. Age at time of completing the MHQ was calculated in years. Gender was self-reported biological sex (female/male). Given the majority of the sample (97%) reported their ethnic background to be ‘White’ or ‘White British’, we dichotomised ethnic background into two groups for comparison: ‘White British’ and ‘UK ethnic minority backgrounds’. The UK ethnic minority backgrounds group included individuals who identified as ‘Black or Black British’ (0.6%; including Caribbean and African backgrounds), ‘mixed background’ (0.6%; including White and Black Caribbean, White and Black African, White and Asian, and other mixed ethnic backgrounds), ‘Chinese’ (0.2%), ‘Asian’ (0.7%; including Indian, Pakistani, Bangladeshi and other Asian backgrounds) and ‘other background’ (0.6%). We acknowledge that pooling individuals under one label is a limitation of the study. However, this was done to preserve statistical power while retaining all individuals for analysis. We used the label ‘UK ethnic minority backgrounds’ simply to reflect the fact that the largest proportion of individuals in the UK population identified as White British. Educational attainment was coded using six categorical responses (‘Secondary’ denotes completion of compulsory secondary education, i.e. GCSE level; ‘Further’ refers to completion of further education, i.e. A-levels; ‘Vocational’ includes a range of vocational and professional qualifications and ‘University degree’ denotes a university-level education). We used the most common of these categories (university degree) as the reference category in our analyses. Annual household income was provided as five categorical responses (<£18 000, £18 000–£30 000, £30 000–£52 000, £52 000–£100 000, >£100 000). We used the median category (£30 000–£52 000) as the reference category in our analyses. Neighbourhood deprivation was assessed with the Townsend Deprivation Index (TDI).^18^ TDI scores are regional and incorporate four variables derived from census data (unemployment, car ownership, homeownership and household overcrowding), which are each standardised (mean 0, s.d. 1) and summed to give a total score. Areas with TDI > 0 are more deprived than average, whereas areas with TDI < 0 are more affluent. Social isolation (yes/no) was assigned to participants who reported that they lived alone and did not participate in any social activities or received visits from friends or family less than once per month.

### Statistical analysis

Logistic regression models were fitted using glm function in R version 3.6.3 for macOS (https://cran.r-project.org/bin/macosx/). Based on prior literature, we selected demographic and socioeconomic factors as explanatory variables, and two variables assessing alternative coping strategies (Table 1). Symptom severity scores were included as covariates. All variables were included simultaneously to derive adjusted odds ratios and 95% confidence intervals describing the extent to which these factors are associated with treatment-seeking or treatment receipt when all other variables in the model were held constant. Before analysis, the characteristics of participants with complete versus missing data were compared. Several variables differed statistically between complete cases and those with missingness (Supplementary Tables 1 and 2). As data was unlikely to be missing at random, multiple imputation was not appropriate. To reduce the impact of missing data on findings, missing data indicators were dummy-coded and included in analyses so that all participants were retained for analysis. Effect sizes from this primary model were compared with those estimated in complete case univariable and multivariable models. We also stratified analyses by gender, to investigate whether there are gender-specific sociodemographic factors. Variables with statistically significant gender differences were also entered into a multivariable model that included all of the original variables, plus the relevant variable by gender interaction terms. Effect sizes were compared with two-sample *z*-tests. To assess the strength of evidence for association, a Bonferroni *P*-value was calculated with the number of effectively independent tests, which was computed as the number of principal components that explained 99.5% of the variance in the correlation matrix of all explanatory variables. Variance inflation factors were computed to assess multicollinearity in the multivariable regression models. None of the variables selected for analysis were strongly correlated (maximum variance inflation factor 1.14, maximum *r* = 0.34; Supplementary Table 7).

**Table 1:** Analysis variables included in logistic regression analyses examining associations with treatment-seeking and treatment receipt in a subsample of the UK Biobank participants meeting criteria for lifetime generalised anxiety or major depressive disorder

	Women (*n* = 30 248)	Men (*n* = 14 562)	Total (*N* = 44 810)
Age, years			
Mean (s.d.)	62.1 (7.5)	62.7 (7.8)	62.3 (7.6)
Median	62.0	63.0	63.0
Range	46.0–80.0	46.0–80.0	46.0–80.0
UK ethnic minority			
No	29 360 (97.4%)	14 111 (97.4%)	43 471 (97.4%)
Yes	794 (2.6%)	375 (2.6%)	1169 (2.6%)
Missing data	94	76	170
Annual household income			
<£18 000	4767 (17.7%)	2035 (15.0%)	6802 (16.8%)
£18 000–£30 000	6750 (25.1%)	2927 (21.6%)	9677 (23.9%)
£30 000-£52 000	7656 (28.4%)	3937 (29.1%)	11 593 (28.6%)
£52 000-£100 000	6227 (23.1%)	3634 (26.8%)	9861 (24.4%)
>£100 000	1515 (5.6%)	1017 (7.5%)	2532 (6.3%)
Missing data	3333	1012	4345
Educational attainment			
Secondary	7501 (26.6%)	3121 (23.2%)	10 622 (25.5%)
Further	4556 (16.1%)	1859 (13.8%)	6415 (15.4%)
Vocational	2655 (9.4%)	1600 (11.9%)	4255 (10.2%)
Degree	13 512 (47.9%)	6859 (51.0%)	20 371 (48.9%)
Missing data	2024	1123	3147
Neighbourhood deprivation			
Mean (s.d.)	−1.4 (2.9)	−1.3 (3.1)	−1.4 (3.0)
Median	−2.1	−2.1	−2.1
Range	−6.3 to 11.0	−6.3 to 10.5	−6.3 to 11.0
Missing data	48	30	78
Social isolation			
No	26 992 (90.5%)	12 649 (88.2%)	39 641 (89.8%)
Yes	2826 (9.5%)	1700 (11.8%)	4526 (10.2%)
Missing data	430	213	643
Self-help			
No	21 155 (69.9%)	11 676 (80.2%)	32 831 (73.3%)
Yes	9093 (30.1%)	2886 (19.8%)	11 979 (26.7%)
Self-medication with alcohol or drugs			
No	25 306 (83.7%)	10 950 (75.2%)	36 256 (80.9%)
Yes	4942 (16.3%)	3612 (24.8%)	8554 (19.1%)
Lifetime symptom severity			
Mean (s.d.)	2.3 (0.6)	2.3 (0.6)	2.3 (0.6)
Median	2.4	2.4	2.4
Range	0.0–3.0	0.0–3.0	0.0–3.0
Missing data	13	5	18

Data are displayed as *n* (%), unless otherwise indicated. Neighbourhood deprivation is measured using the Townsend Deprivation Index. Here, positive scores reflect greater neighbourhood deprivation than the UK average (0). The analysis sample has a negative mean deprivation score, indicating that on average this sample is from more affluent neighbourhoods than the average UK population. Lifetime symptom severity score is calculated as the proportion of Composite International Diagnostic Interview Short-Form symptoms endorsed, weighted by overall functional impairment; scores were standardised to have a mean of 0 and s.d. of 1 in all analyses

## Results

### Characteristics of the analysis sample

There were 44 810 participants (age: mean 62.3 years, s.d. 7.6 years) with complete data on treatment-seeking and 37 346 participants with data for treatment receipt (Supplementary Figure 1). In total, 7064 participants had missing data on at least one analysis variable. Compared with participants with missing data, those with complete data were younger (mean 61.8 *v*. 65.2 years, *P* < 0.001; Supplementary Table 2), more likely to be male (33.1 *v*. 29.3%, *P* < 0.001) and more likely to have a university degree (50.0 *v*. 37.8%, *P* < 0.001), but were less likely to have a lower household income (annual household income < £18 000: 14.9 *v*. 44.0%, *P* < 0.001) and be from less deprived neighbourhoods (−1.4 *v*. −1.2, *P* < 0.001). They were more likely to have used self-help strategies (27.6 *v*. 22.0%, *P* < 0.001) and self-medication with alcohol/drugs (19.9 *v*. 14.8%, *P* < 0.001). More participants with complete data reported seeking treatment than those with missing data (84.2 *v*. 78.7%, *P* < 0.001). There was no evidence for differences in treatment receipt between the analytical sample and those with missing data (Supplementary Table 1).

### The treatment gap

Overall, 83.3% had sought professional help for their symptoms (Table 2). Of these, 89.2% had received treatment. Therefore, 74.4% of those who met criteria for a lifetime diagnosis had both sought and received treatment. The proportion of cases in this sample who had never sought or received treatment, defined as the treatment gap, was 25.6%. The majority of the treatment gap (16.7%) comprised those who never sought treatment, defined here as the seeking gap. The access gap, the proportion of cases who seek treatment but do not go on to receive it, comprised the remaining 9% (Supplementary Figure 2).

**Table 2:** Treatment-seeking and treatment receipt outcomes in a subsample of the UK Biobank participants meeting criteria for lifetime generalised anxiety or major depressive disorder

	Women (*n*=30 248)	Men (*n* = 14 562)	Total (*N* = 44 810)
Treatment-seeking			
No	4302 (14.2%)	3163 (21.7%)	7465 (16.7%)
Yes	25 946 (85.8%)	11 399 (78.3%)	37 345 (83.3%)
Treatment receipt			
No	2637 (10.2%)	1387 (12.2%)	4024 (10.8%)
Yes	23 309 (89.8%)	10 012 (87.8%)	33 321 (89.2%)
Missing data	4302	3163	7465
Total cases treated			
No	6939 (22.9%)	4550 (31.2%)	11 489 (25.6%)
Yes	23 309 (77.1%)	10 012 (68.8%)	33 321 (74.4%)

### Factors associated with treatment-seeking

**Fig. 1:**
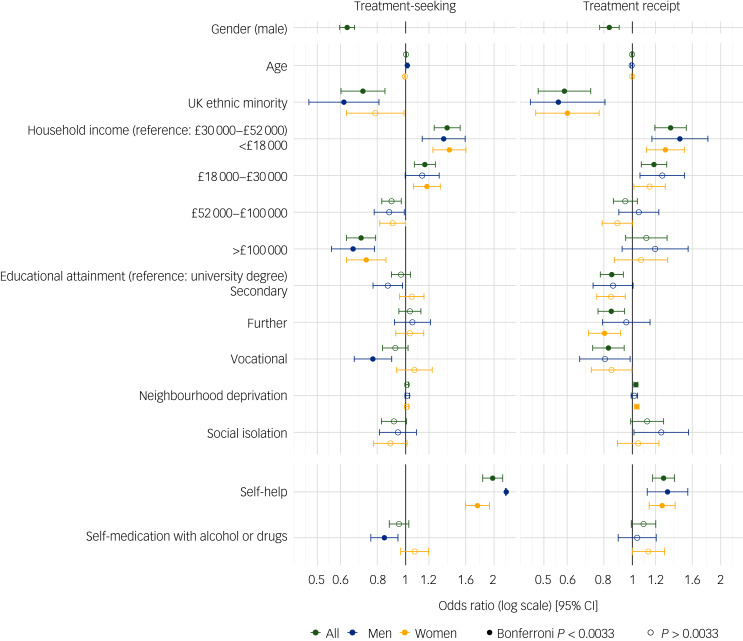
Factors associated with treatment seeking and receipt in UK Biobank participants meeting criteria for lifetime generalised anxiety or major depressive disorder. Odds ratios, 95% confidence intervals and *P*-values estimated from multivariable regression analyses of treatment seeking and treatment receipt in the full sample (green; *n* = 44 810 and 37 346), in males (blue; *n* = 10 737 and 8 733) and in females (yellow; *n* = 22 967 and 20 207).

Effect sizes and confidence intervals are reported in Table 3 and displayed in Figure 1. Being male was the strongest sociodemographic factor associated with treatment-seeking, whereby men had lower odds of seeking treatment than women (odds ratio 0.63, 95% CI 0.60–0.67; Figure 1, Table 3). Participants from UK ethnic minority backgrounds were also less likely to seek treatment than those identifying as White British (odds ratio 0.72, 95% CI 0.60–0.85). Compared with those in the median annual household income group (£30 000–£52 000), those with lower incomes were more likely to seek treatment and those with higher incomes were less likely to seek treatment (e.g. annual household income < £18 000: odds ratio 1.38, 95% CI 1.25–1.53; annual household income >£100 000: odds ratio 0.70, 95% CI 0.63–0.79). Self-help behaviour was the strongest association with treatment-seeking from a professional (odds ratio 1.98, 95% CI 1.83–2.14). When we compared effects estimated in the multivariable model with univariable models, the only notable difference in effect size was for self-medication, whereby the univariable effect had an opposite direction of effect compared with the multivariable model (multivariable odds ratio 0.95, univariable odds ratio 1.12, *Z*_difference_ = −3.87, *P*_difference_ = 1.00 × 10^−3^; Supplementary Figure 4, Supplementary Table 5).

**Table 3:** Factors associated with treatment-seeking and treatment receipt in UK Biobank participants meeting CIDI-SF criteria for a lifetime diagnosis of generalised anxiety or major depressive disorder

	Treatment-seeking			Treatment receipt		
Variable	Odds ratio	95% CI	*P*-value	Odds ratio	95% CI	*P-*value
Age (per year)	1.00	[1 to 1.01]	1.93e-01	1.00	[0.99 to 1]	3.84e-01
Gender (reference: Female)						
Male	0.63	[0.6 to 0.67]	6.80e-52	0.84	[0.77 to 0.9]	4.21e-06
UK ethnic minority (reference: no)						
Yes	0.72	[0.6 to 0.85]	1.37e-04	0.59	[0.48 to 0.72]	3.80e-07
Missing data	0.63	[0.39 to 1.04]	6.98e-02	0.78	[0.4 to 1.51]	4.55e-01
Annual household income (reference: £30 000–£52 000)						
<£18 000	1.38	[1.25 to 1.53]	5.70e-10	1.35	[1.19 to 1.53]	1.79e-06
£18 000–£30 000	1.16	[1.07 to 1.26]	3.69e-04	1.19	[1.07 to 1.31]	8.67e-04
£52 000–£100 000	0.90	[0.83 to 0.97]	4.32e-03	0.95	[0.86 to 1.04]	2.57e-01
>£100 000	0.70	[0.63 to 0.79]	1.80e-09	1.12	[0.95 to 1.31]	1.86e-01
Educational attainment (reference: university degree)						
Secondary	0.96	[0.9 to 1.04]	3.27e-01	0.85	[0.78 to 0.93]	5.12e-04
Further	1.03	[0.95 to 1.13]	4.52e-01	0.85	[0.76 to 0.94]	1.92e-03
Vocational	0.92	[0.83 to 1.02]	1.13e-01	0.83	[0.73 to 0.94]	3.04e-03
Neighbourhood deprivation (per s.d.)	1.01	[1 to 1.02]	9.01e-02	1.03	[1.01 to 1.04]	1.52e-04
Social isolation (reference: no)						
Yes	0.91	[0.83 to 1.01]	6.49e-02	1.12	[0.99 to 1.28]	7.71e-02
Missing data	0.78	[0.48 to 1.28]	3.26e-01	1.54	[0.71 to 3.35]	2.71e-01
Self-help (reference: no)						
Yes	1.98	[1.83 to 2.14]	1.00e-66	1.28	[1.17 to 1.39]	1.92e-08
Self-medication with alcohol or drugs (reference: no)						
Yes	0.95	[0.88 to 1.02]	1.82e-01	1.09	[0.99 to 1.2]	7.34e-02

Odds ratios, 95% confidence intervals and *P*-values estimated from multivariable regression analyses. CIDI-SF, Composite International Diagnostic Interview Short-Form.

In stratified samples**,** 14 562 men and 30 248 women had data for treatment-seeking analyses. Similar to unstratified analyses, being from an ethnic minority group was associated with reduced odds of treatment-seeking in men and women (men: odds ratio 0.62, 95% CI 0.47–0.81; women: odds ratio 0.79, 95% CI 0.63–0.99). Annual household income was also negatively associated with treatment-seeking in both men and women: for annual household income <£18 000, we found an odds ratio of 1.35 (95% CI 1.14–1.59) for men and 1.41 (95% CI 1.24–1.60) for women; and for annual household income >£100 000, we found an odds ratio of 0.66 (95% CI 0.56–0.78) for men and 0.73 (95% CI 0.63–0.86) for women.

There were four statistically significant gender differences in effect sizes for self-medication, self-help, educational attainment and age (Figure 1, Supplementary Table 5). Self-medication with alcohol was associated with lower odds of treatment-seeking in men, but not in women (men: odds ratio 0.85, 95% CI 0.76–0.94; women: odds ratio 1.07, 95% CI 0.96–1.20; *Z*_difference_ = 3.05; *P*_difference_ = 2.29 × 10^−3^). Self-help behaviour was positively associated with treatment-seeking in both genders, but had a larger effect in men than women (men: odds ratio 2.51, 95% CI 2.19–2.89; women: odds ratio 1.76, 95% CI 1.60–1.93; *Z*_difference_ = −4.18; *P*_difference_ = 2.92 × 10^−5^). Only one educational effect had gender differences, which was the comparison between having a university degree and a vocational qualification. Here, men who had a vocational qualification were less likely to seek treatment (odds ratio 0.77, 95% CI 0.67–0.90), whereas there was no clear effect of having a vocational qualification for women (odds ratio 1.07, 95% CI 0.93–1.23; *Z*_difference_ = 3.16; *P*_difference_ = 1.58 × 10^−3^). Age showed no association with treatment-seeking in the full sample, but showed opposite effects between gender. Older men were more likely to have sought treatment than younger men (odds ratio 1.01 per year, 95% CI 1.01–1.02), but younger women were more likely to have sought treatment than older women (odds ratio 0.99 per year, 95% CI 0.99–1.00; *Z*_difference_ = −4.48; *P*_difference_ = 7.50 × 10^−6^). Given that age, educational attainment, self-help and self-medication had statistically significant gender differences, we ran an additional multivariable model, which included these variable×gender interaction terms. Estimates from this model are provided in Supplementary Table 7.

### Factors associated with treatment receipt

Of treatment-seekers (*n* = 37 346), 89.2% reported receiving treatment. The strongest factor associated with treatment receipt was ethnicity. Specifically, individuals from ethnic minority groups in the UK were less likely to receive treatment than those of White British ethnicity (odds ratio 0.59, 95% CI 0.48–0.72; Figure 1, Table 3). Male gender was also associated with lower odds of receiving treatment (odds ratio 0.84, 95% CI 0.77–0.90). Compared with having a university degree, having completed secondary education, further education and vocational qualification were all associated with lower odds of receiving treatment (odds ratio 0.83–0.85, 95% CI_[minimum, maximum]_ 0.73–0.94). Factors associated with increased odds of treatment receipt were lower than median annual household income (odds ratio 1.19–1.35, 95% CI_[minimum, maximum]_ 1.07–1.53), neighbourhood deprivation (odds ratio 1.03 per unit change, 95% CI 1.01–1.04) and use of self-help behaviour strategies (odds ratio 1.28, 95% CI 1.17–1.39). There were no notable differences in effect sizes between multivariable and univariable models (Supplementary Table 5).

In stratified analyses of treatment receipt**,** there were 11 399 men and 25 946 women. Similar to the main analysis, factors associated with treatment receipt in women included ethnicity (odds ratio 0.60, 95% CI 0.47–0.77), educational attainment other than a university degree (odds ratio 0.83, 95% CI_[minimum, maximum]_ 0.66–1.00) and neighbourhood deprivation (odds ratio 1.04, 95% CI 1.02–1.05). Although these effects were of similar size in men, for some, the strength of evidence was weaker (ethnicity: odds ratio 0.68, 95% CI 0.39–0.85; education: odds ratio 0.86, 95% CI 0.75–0.99; neighbourhood deprivation: odds ratio 1.01, 95% CI 0.99–1.04). This is likely because stratifying the sample by gender resulted in a substantial difference in sample size and statistical power between strata. However, there were no gender differences in factors associated with treatment receipt (maximum *Z*_difference_ = −1.94; *P*_difference_ = 0.05; Supplementary Table 5).

## Discussion

### Main findings

We estimated the proportion of UK Biobank participants who never sought or received treatment despite meeting criteria for lifetime anxiety or depressive disorder diagnosis, defined here as the lifetime treatment gap. In this sample, the lifetime treatment gap was 25.6%. This estimate reflects a lack of treatment access over the life course. In contrast, previous point prevalence estimates of 60–70%^1,19^ were obtained from studying shorter time intervals, which are more likely to be within the context of a single period of poor mental health. Notably, participants in the UK Biobank are middle-aged or older, and therefore are more likely to have received treatment at some point in their lives than younger adults. Furthermore, the socioeconomic circumstances of the UK Biobank participants are more advantaged than the general population, which may also explain differences between the estimates. We also reported factors associated with lifetime treatment-seeking and treatment receipt. In these data, being male or from an ethnic minority group in the UK context were the strongest sociodemographic factors associated with treatment-seeking and treatment receipt, respectively.^20^ These factors have been associated with treatment receipt at the time of treatment need.^1^

Gender differences in treatment-seeking for mental health problems have been widely discussed. Being male is associated with negative treatment-seeking attitudes^21^ and low willingness to seek treatment.^22^ Likely mediators of these attitudes include male socialisation, masculine traits and associated self-stigma,^23,24^ poor mental health literacy^25^ and differences in coping strategies (e.g. higher likelihood to self-medicate with alcohol/drugs).^12,26^ When we stratified our analyses by gender, the most notable gender differences were the effects of alternative coping strategies. Men who reported self-medicating with alcohol/drugs were less likely to have sought treatment for their anxiety or depression. Alcohol use problems and MDD commonly co-occur.^27^ Indeed, the participants of this study who reported self-medicating with alcohol/drugs could have also had co-occurring substance-related problems. More work is required to better understand the relationship between depression, self-medication and substance related problems. If treatment receipt for depression moderates the relationship between depression and alcohol use problems, improving treatment-seeking and treatment receipt for anxiety and depression could be an important target for reducing the collective burden from these disorders.^28^ Overall, reported use of a self-help strategy was the strongest predictor of treatment-seeking. This positive effect was largest in men. However, it is difficult to determine the direction of effect. Use of self-help strategies might reflect good mental health literacy and increased likelihood of seeking treatment. Equally, self-help activities are often prescribed by clinicians. Nonetheless, it could suggest a general willingness to seek and try out an intervention. Preliminary research suggests those who struggle more with stigma are more likely to engage with online self-help interventions than to seek help.^29,30^ Such resources may act as a gateway to professional help. Educational interventions have been shown to improve treatment-seeking attitudes and behaviours, specifically in men.^9^

Individuals from UK minority ethnic groups were also less likely to seek and receive treatment than White British participants in this study. Qualitative work with participants from UK minority ethnic groups identified personal–environmental factors (health literacy, social support, culture and associated stigma) and patient–provider relationship factors (wait times, communication difficulties and discrimination) as barriers to treatment for them.^10^ More work is required to understand the role of cultural, systematic and clinician factors on the detection and appropriate treatment of mental health problems for individuals exposed to minority stress and factors specific to different ethnic groups. The small proportion of participants from different ethnic groups in our sample meant we were unable to investigate the differences between different ethnic groups or the specific socioeconomic factors associated with treatment receipt, which is a key limitation of the study. Psychosocial barriers are likely to be the mediating factors between sociodemographic factors and treatment-seeking and treatment receipt. In the UK, research shows these barriers include stigmatising attitudes toward mental health problems and treatments, lack of trust in current treatments^4,5^ and perceived clinician biases.^10^ In our analysis, men and individuals from ethnic minority groups in the UK are at risk of not accessing treatment, and this may be a result of them experiencing these barriers.

With regard to socioeconomic factors, a more complex pattern emerged. Higher income was associated with reduced odds of treatment-seeking and treatment receipt and neighbourhood deprivation was associated with increased odds of treatment receipt. The finding that more affluent individuals were less likely to seek and receive treatment is surprising. Previous work shows that although those with more advantageous socioeconomic circumstances are less likely to seek treatment overall, they are more likely to receive it if they do seek it **–** referred to as the ‘inverse care law’.^11,31^ The difference observed here may reflect the high sociodemographic profile of this sample compared with the general population of the UK, as those from less advantaged socioeconomic backgrounds are not well-represented in the sample. It might also be the case that more affluent participants in our sample have stronger social support networks, which helps to alleviate symptoms and the need for treatment.^32^ Compared with having a university-level education, secondary, further and vocational educational levels were all associated with lower odds of receiving treatment after having sought it. University-level education is associated with improved mental health literacy and reduced stigmatising attitudes toward mental health conditions and treatments.^33,34^ Therefore, those with university level education might be better equipped to recognise and communicate their symptoms and needs effectively, or more open to engaging with treatments.

### Limitations

The characteristics of the UK Biobank sample and the cross-sectional study design mean that interpretations of these findings should be made with caution. Selection bias in the context of population-based cohort studies in general, and in the UK Biobank specifically, is a key limitation that has been widely discussed.^14–16^ Especially relevant for this study, the subsample of UK Biobank participants who completed the follow-up MHQ have more advantaged socioeconomic circumstances, including higher educational attainment, than the general UK population.^14^ Participation was also negatively associated with being male, having mental or physical disorder diagnoses, being a smoker and having a higher body mass index, and positively associated with having a university degree and having a relative who has had severe depression.^16^ Importantly, some of these factors are also associated with access to treatment,^3,11^ and were associated with treatment-seeking or treatment receipt in our analyses. However, although the lack of representativeness has been shown to bias estimates of prevalence, analyses using large samples containing such biases have been shown to produce risk factor associations with outcomes that are generalisable to the population under study.^15,35^ For example, cardiovascular-related risk factor associations in the UK Biobank and a UK representative sample (Health Surveys for England and the Scottish Health Surveys consortium) had an overall effect size ratio close to unity, despite the differences in sample selection.^36^ Although associations detected in non-random samples should be interpreted with caution, where data on outcomes of interest exists in large biobank data-sets, exploratory analyses such as ours can be used to inform future analyses and replication efforts in representative samples.

Our focus on lifetime outcomes allowed us to detect factors associated with treatment-seeking and treatment receipt that are potentially pervasive across the lifespan. However, our analysis was cross-sectional and the sample was age variable. It is reasonable to expect that the probability of seeking treatment would be higher in older participants, because of the increased probability of an event over time, yet we did not observe an effect of age on access to treatment. This is potentially because of changes in the availability and knowledge of mental healthcare over time, or recall difficulties in older participants. The participants included in this analysis were older than the general population, and it is likely some will have experienced their symptoms earlier in life, when both mental health literacy and access to treatment were less common than they are now. It is also plausible that treatment undertaken earlier in life for older participants would not be as readily recalled as more recent treatment undertaken by younger participants. The potentially opposite effects of ‘probability of access over time’ and ‘change in resources over time’ could potentially induce a cohort effect, and therefore, future analyses of treatment access may benefit from stratification by age or longitudinal study designs.

The clinical utility of public health interventions aiming to predict or encourage treatment-seeking relies on several assumptions. One is that such interventions are effective. However, there is mixed evidence in support of help-seeking interventions. A systematic review found that although such initiatives had small effects on improving treatment-seeking attitudes, they had no effect on treatment-seeking behaviour.^37^ Therefore, more work is required to improve the efficacy of treatment-seeking interventions, and to assess the long term benefits following them. Another assumption is that everyone who experiences symptoms needs and will benefit from treatment, and furthermore, that healthcare services have capacity to see and treat more patients. Many individuals with anxiety and depression spontaneously remit over time,^38^ some who receive treatment do not respond.^39^ Further, for those who do seek psychological therapies, there are often long wait-lists, the length of which has been shown to predict poorer outcomes.^40^ Therefore, efforts to improve treatment-seeking must also be matched with increased availability of treatment, and such efforts need to be targeted at those who are unlikely to remit without intervention.

### Future directions

Longitudinal studies are needed to assess whether treatment-seeking and treatment receipt moderate trajectories of symptoms over time, as well as educational, occupational and life outcomes, to determine how effective interventions are likely to be. Educational and occupational outcomes could be assessed in longitudinal cohorts of children and adolescents, and later-life outcomes assessed in older adult samples. Later-life outcomes could include long-term prognosis (i.e. course and chronicity of symptoms), additional mental and physical diagnoses, and negative outcomes such as alcohol and substance use disorders and suicide. Although there was information available on the broad category of treatment received (medication and/or talking therapy), we focused on the receipt of any kind of treatment. Future studies could investigate treatment access in more detail.

Our findings suggest that out of all of the individuals who reported sufficient number and burden of anxiety or depressive symptoms to meet diagnostic criteria, men and individuals from ethnic minority backgrounds were less likely to access treatment. Psychosocial barriers are likely to be the mediating factors between sociodemographic variables and treatment-seeking and treatment receipt. In the UK, research shows that these barriers include stigmatising attitudes toward mental health problems and treatments, lack of trust in current treatments^4,5^ and perceived clinician biases.^10^ Men and individuals from ethnic minority backgrounds are at increased risk of not accessing treatment in our analyses, and this may be because they are experiencing these barriers more frequently. This includes not receiving treatment even after having sought it. Thus, clinicians also have an important role to play in reducing the access gap (9% in this sample), by offering targeted information and support to treatment-seeking individuals who are at risk of experiencing stigma or discrimination. Much work is needed to investigate barriers to treatment for marginalised populations, as research grouping participants together is not sufficient to make evidence-based conclusions to adequately serve the many groups. In conclusion, the current findings represent an important step toward identifying groups of individuals at risk of not seeking or receiving treatment for anxiety and depression. Future work to investigate both the specific psychosocial factors that mediate these associations, and targeted interventions attempting to address them, are likely to yield significant benefit for individuals, families and society, by reducing the burden of untreated anxiety and depression.

## Data Availability

Data is available from UK Biobank subject to standard procedures (www.ukbiobank.ac.uk). Analysis code for replication is available from GitHub (https://github.com/chrisrayner).

## Supplementary material

For supplementary material accompanying this paper visit http://dx.doi.org/10.1192/bjo.2021.1012. click here to view supplementary material
