# Supplementary material for: Sociodemographic factors associated with treatment-seeking and treatment receipt: cross-sectional analysis of UK Biobank participants with lifetime generalised anxiety or major depressive disorder
Source: BJPsych Open. 2021 Nov 19;7(6):e216. doi: 10.1192/bjo.2021.1012 (PMC8612017; doi:10.1192/bjo.2021.1012)
Supplement: Supplementary file 1 [file S2056472421010127sup001.docx]

Supplement: Sociodemographic factors associated with treatment seeking and receipt for common mental health problems in the UK Biobank

Rayner et al. 2020

Table of Contents

[Supplementary Methods 1](#_Toc77160071)

[S.Fig 1. Participants 1](#_Toc77160072)

[Phenotypes 3](#_Toc77160073)

[Statistical Analyses 4](#_Toc77160074)

[Supplementary Results 5](#_Toc77160075)

[S.Tables 5](#_Toc77160076)

[S.Table 1.Outcomes 5](#_Toc77160077)

[S.Table 2. Analysis variables 6](#_Toc77160078)

[S.Table 3. Correlations 9](#_Toc77160079)

[S.Table 4. Regression analyses 11](#_Toc77160080)

[S.Table 5. Two-sample Z-tests 14](#_Toc77160081)

[S.Table 6. VIFS 16](#_Toc77160082)

[S.Figures 17](#_Toc77160083)

[S.Fig 2. The gaps 17](#_Toc77160084)

[S.Fig 3. Odds ratios 18](#_Toc77160085)

# Supplementary Methods

## S.Fig 1. Participants

**S.Fig 1. Flowchart of UK Biobank participants included in analyses**


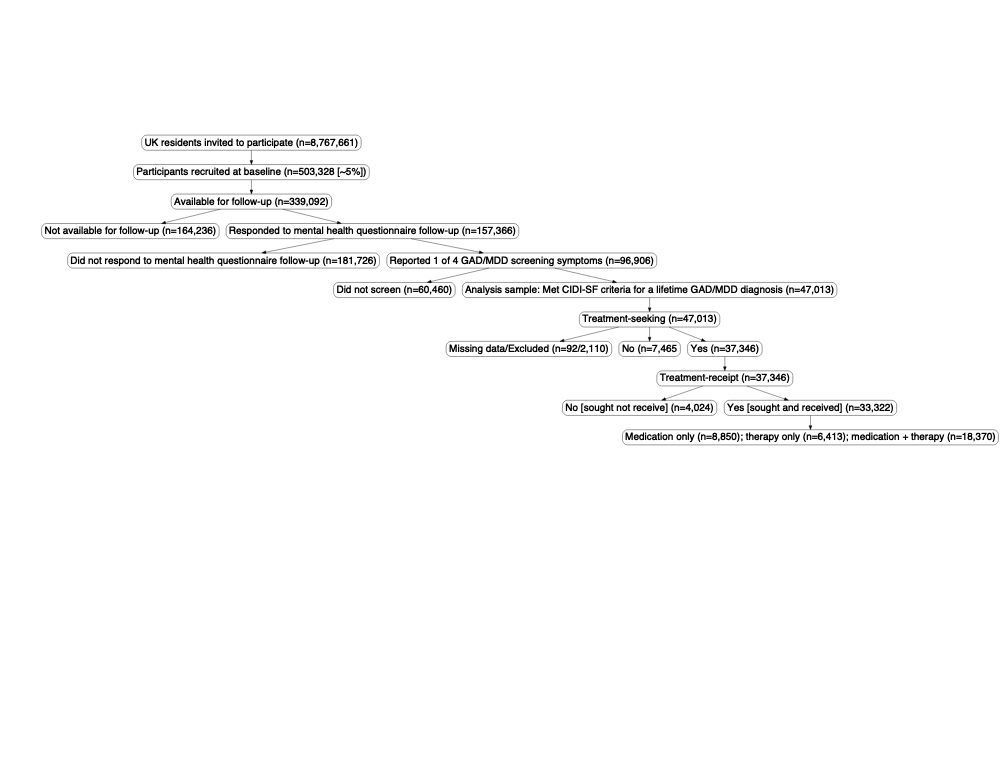


## Phenotypes

**Diagnostic criteria**

Participants were assessed as having lifetime **generalised anxiety disorder** if they reported experience of the following CIDI-SF symptoms:

1. **EITHER** Having worried more than most people would in the same situation [f.20425] **OR** have had stronger worry than most people during their worst period of anxiety [f.20542].
2. Have had at least one time in their lives where they have felt worried, tense, or anxious most of the time for at least a month [f.20421]
3. This frequent and persistent worrying has persisted 6 months or more [f.20420]
4. Report having worried most days during their worst period of anxiety [f.20538]
5. **EITHER** had many different worries on their mind at the same time during their worst period [f.20540] **OR** worried about more than one thing during the worst period of anxiety [f.20543].
6. Found it difficult to stop worrying [f.20541] OR were often unable to stop worrying [f.20539] OR often found themselves unable to control their worry [f.20537].
7. Suffered 3 or more somatic symptoms of anxiety during the worst period of anxiety (these include feelings of restlessness [f.20426]; feeling easily tired [f.20429]; having trouble falling or staying asleep [f.20427]; feeling keyed up or on edge [f.20423]; increased irritability [f.20422]; experiencing tense, sore or aching muscles [f.20417]; having difficulty concentrating [f.20419])
8. Their anxiety resulted in significant impairment in roles or normal function [f.20418]

Participants were assessed as having lifetime **major depressive disorder** if they reported experience of the following symptoms:

1. **EITHER** Prolonged loss of interest or pleasure in doing things [f.20514] **OR** Prolonged feelings of depression [f.20510]
2. Fraction of day affected being most of the day during worst episode of depression [f.20436]
3. Frequency of depressed days during worst episode of depression being almost every day (or more) [f.20439]
4. At least a moderate impact on normal roles during the worst period of depression [f.20440]
5. Suffered a total of 5 or more symptoms of major depressive disorder (these included: Prolonged loss of interest or pleasure in doing things [f.20514]; Prolonged feelings of depression [f.20510]; Feelings of tiredness or low energy [f.20519]; Weight change [f.20536]; Trouble falling or staying asleep, or sleeping too much [f.20517]; Difficulty concentrating on things [f.20508]; Feelings of worthlessness [f.20450]; Thoughts of death [f.20513]

## Statistical Analyses

**Variance Inflation Factors**

To calculate VIFs, first, each variable in the analytical model is included as the dependent variable in a multiple regression, which includes all other explanatory variables from the model. The variance of each explanatory variable (j) that is explained by all other explanatory variables in the model (R2j) is used to calculate the VIF as follows: VIFj=1/(1-R2j) 18. As such, the VIF indicates the degree to which an estimated regression coefficient is increased because of collinearity. For example, a VIF of 1.90 indicates that the variable’s effect size is inflated by 90% because of its correlations with other variables in the model. A VIF value of 1 indicates no correlation and typically, values of 1 to 5 indicate moderate collinearity and a value greater than 5 indicates that the variable is highly correlated with other variables in the model, although “problematic” VIF values vary by the type of analysis 19.

# Supplementary Results

## S.Tables

### S.Table 1.Outcomes

**S.Table 1. Treatment-seeking and receipt outcomes** in a subsample of the UK Biobank participants meeting criteria for lifetime generalised anxiety or major depressive disorder. Participants are stratified into complete-cases and those with missing data on one or more of the analysis variables. Group differences were compared using chi-square tests

|  | Complete data (N=37746) | Missing data (N=7064) | Total (N=44810) | p value |
| --- | --- | --- | --- | --- |
| **Treatment-seeking** |  |  |  | < 0.001^1^ |
| No | 5960 (15.8%) | 1505 (21.3%) | 7465 (16.7%) |  |
| Yes | 31786 (84.2%) | 5559 (78.7%) | 37345 (83.3%) |  |
| **Treatment-receipt** |  |  |  | 0.778^1^ |
| No | 3419 (10.8%) | 605 (10.9%) | 4024 (10.8%) |  |
| Yes | 28367 (89.2%) | 4954 (89.1%) | 33321 (89.2%) |  |
| Missing data | 5960 | 1505 | 7465 |  |
| **Total cases treated** |  |  |  | < 0.001^1^ |
| No | 9379 (24.8%) | 2110 (29.9%) | 11489 (25.6%) |  |
| Yes | 28367 (75.2%) | 4954 (70.1%) | 33321 (74.4%) |  |

### S.Table 2. Analysis variables

**S.Table 2. Analysis variables** included in logistic regression analyses examining associations with treatment-seeking and receipt in a subsample of the UK Biobank participants meeting criteria for lifetime generalised anxiety or major depressive disorder. Participants are stratified into complete-cases and those with missing data on one or more of the analysis variables. Group differences were compared using chi-square or ANOVA tests

|  | Complete data (N=37746) | Missing data (N=7064) | Total  (N=44810) | p value |
| --- | --- | --- | --- | --- |
| **Age** |  |  |  | < 0.001^1^ |
| Mean (SD) | 61.8 (7.6) | 65.2 (7.3) | 62.3 (7.6) |  |
| Median | 62.0 | 66.0 | 63.0 |  |
| Range | 46.0 - 79.0 | 47.0 - 80.0 | 46.0 - 80.0 |  |
| **Sex** |  |  |  | < 0.001^2^ |
| Female | 25255 (66.9%) | 4993 (70.7%) | 30248 (67.5%) |  |
| Male | 12491 (33.1%) | 2071 (29.3%) | 14562 (32.5%) |  |
| **UK ethnic minority** |  |  |  | 0.269^2^ |
| No | 36771 (97.4%) | 6700 (97.2%) | 43471 (97.4%) |  |
| Yes | 975 (2.6%) | 194 (2.8%) | 1169 (2.6%) |  |
| Missing data | 0 | 170 | 170 |  |
| **Household income** |  |  |  | < 0.001^2^ |
| <£18,000 | 5606 (14.9%) | 1196 (44.0%) | 6802 (16.8%) |  |
| £18,000-£30,000 | 8897 (23.6%) | 780 (28.7%) | 9677 (23.9%) |  |
| £30,000-£52,000 | 11097 (29.4%) | 496 (18.2%) | 11593 (28.6%) |  |
| £52,000-£100,000 | 9646 (25.6%) | 215 (7.9%) | 9861 (24.4%) |  |
| >£100,000 | 2500 (6.6%) | 32 (1.2%) | 2532 (6.3%) |  |
| Missing data | 0 | 4345 | 4345 |  |
| **Educational attainment** |  |  |  | < 0.001^2^ |
| Secondary | 9333 (24.7%) | 1289 (32.9%) | 10622 (25.5%) |  |
| Further | 5770 (15.3%) | 645 (16.5%) | 6415 (15.4%) |  |
| Vocational | 3752 (9.9%) | 503 (12.8%) | 4255 (10.2%) |  |
| Degree | 18891 (50.0%) | 1480 (37.8%) | 20371 (48.9%) |  |
| Missing data | 0 | 3147 | 3147 |  |
| **Neighbourhood deprivation** |  |  |  | < 0.001^1^ |
| Mean (SD) | -1.4 (3.0) | -1.2 (3.1) | -1.4 (3.0) |  |
| Median | -2.2 | -2.0 | -2.1 |  |
| Range | -6.3 - 11.0 | -6.3 - 10.2 | -6.3 - 11.0 |  |
| Missing data | 0 | 78 | 78 |  |
| **Social isolation** |  |  |  | 0.119^2^ |
| No | 33913 (89.8%) | 5728 (89.2%) | 39641 (89.8%) |  |
| Yes | 3833 (10.2%) | 693 (10.8%) | 4526 (10.2%) |  |
| Missing data | 0 | 643 | 643 |  |
| **Self-help** |  |  |  | < 0.001^2^ |
| No | 27319 (72.4%) | 5512 (78.0%) | 32831 (73.3%) |  |
| Yes | 10427 (27.6%) | 1552 (22.0%) | 11979 (26.7%) |  |
| **Self-medication with alcohol or drugs** |  |  |  | < 0.001^2^ |
| No | 30234 (80.1%) | 6022 (85.2%) | 36256 (80.9%) |  |
| Yes | 7512 (19.9%) | 1042 (14.8%) | 8554 (19.1%) |  |
| **Lifetime symptom severity** |  |  |  | < 0.001^1^ |
| Mean (SD) | 2.3 (0.6) | 2.3 (0.6) | 2.3 (0.6) |  |
| Median | 2.4 | 2.3 | 2.4 |  |
| Range | 0.0 - 3.0 | 0.0 - 3.0 | 0.0 - 3.0 |  |
| Missing data | 0 | 18 | 18 |  |

### S.Table 3. Correlations between analysis variables

| Variable 1 | Variable 2 | r | se |
| --- | --- | --- | --- |
| University degree | Income | 0.34 | 0.007 |
| Income | Age | -0.27 | 0.006 |
| Neighbourhood deprivation | UK ethnic minority | 0.25 | 0.010 |
| Social isolation | Income | -0.25 | 0.011 |
| UK ethnic minority | Age | -0.23 | 0.012 |
| Self help | Self medication with alcohol or drugs | 0.22 | 0.008 |
| Sex | Self help | -0.20 | 0.008 |
| Social isolation | Neighbourhood deprivation | 0.20 | 0.007 |
| Sex | Self medication with alcohol or drugs | 0.19 | 0.008 |
| Lifetime symptom severity | Self help | 0.17 | 0.006 |
| Neighbourhood deprivation | Income | -0.17 | 0.006 |
| Age | Self medication with alcohol or drugs | -0.16 | 0.006 |
| Lifetime symptom severity | Self medication with alcohol or drugs | 0.14 | 0.007 |
| Age | Self help | -0.14 | 0.006 |
| Neighbourhood deprivation | Self medication with alcohol or drugs | 0.12 | 0.006 |
| University degree | UK ethnic minority | 0.12 | 0.015 |
| University degree | Self help | 0.11 | 0.008 |
| Neighbourhood deprivation | Age | -0.11 | 0.005 |
| Lifetime symptom severity | Age | -0.10 | 0.005 |
| Social isolation | UK ethnic minority | 0.10 | 0.019 |
| Income | Sex | 0.09 | 0.008 |
| Social isolation | Sex | 0.08 | 0.010 |
| Social isolation | Self medication with alcohol or drugs | 0.07 | 0.011 |
| Income | Self medication with alcohol or drugs | 0.05 | 0.009 |
| University degree | Self medication with alcohol or drugs | 0.05 | 0.009 |
| UK ethnic minority | Self help | 0.05 | 0.016 |
| Lifetime symptom severity | Income | -0.05 | 0.006 |
| Neighbourhood deprivation | Self help | 0.04 | 0.006 |
| Sex | Age | 0.04 | 0.006 |
| University degree | Age | -0.04 | 0.006 |
| University degree | Sex | 0.04 | 0.008 |
| Income | UK ethnic minority | -0.04 | 0.017 |
| Lifetime symptom severity | Neighbourhood deprivation | 0.04 | 0.005 |
| Lifetime symptom severity | Social isolation | 0.04 | 0.008 |
| UK ethnic minority | Self medication with alcohol or drugs | -0.03 | 0.018 |
| Income | Self help | 0.03 | 0.009 |
| Lifetime symptom severity | UK ethnic minority | 0.03 | 0.012 |
| Neighbourhood deprivation | University degree | 0.03 | 0.006 |
| Social isolation | Self help | -0.02 | 0.011 |
| Neighbourhood deprivation | Sex | 0.02 | 0.006 |
| Lifetime symptom severity | Sex | -0.02 | 0.006 |
| Social isolation | Age | -0.01 | 0.008 |
| UK ethnic minority | Sex | -0.01 | 0.016 |
| Social isolation | University degree | -0.01 | 0.010 |
| Lifetime symptom severity | University degree | -0.01 | 0.006 |

###

### S.Table 4. Regression analyses

**S.Table 4. Summary statistics from multi- and univaribale regression analyses testing for associations between treatment-seeking, treatment receipt and sociodemographic variables**

| **Treatment seeking** | **Full sample** | | | **Male sample** | | | **Female sample** | | |
| --- | --- | --- | --- | --- | --- | --- | --- | --- | --- |
| Variable | OR | 95% CI | P | OR | 95% CI | P | OR | 95% CI | P |
| **Age** (per year) | 1.00 | [1,1.01] | 1.93e-01 | 1.01 | [1.01,1.02] | 2.08e-05 | 0.99 | [0.99,1] | 3.71e-02 |
| **Sex** (ref: Female) |  |  |  |  |  |  |  |  |  |
| Male | 0.63 | [0.6,0.67] | 6.80e-52 | NA | NA | NA | NA | NA | NA |
| **UK ethnic minority** (ref: No) |  |  |  |  |  |  |  |  |  |
| Yes | 0.72 | [0.6,0.85] | 1.37e-04 | 0.62 | [0.47,0.81] | 5.24e-04 | 0.79 | [0.63,0.99] | 3.82e-02 |
| Missing data | 0.63 | [0.39,1.04] | 6.98e-02 | 0.76 | [0.39,1.51] | 4.37e-01 | 0.50 | [0.24,1.01] | 5.28e-02 |
| **Household income** (ref: £30,000-£52,000) |  |  |  |  |  |  |  |  |  |
| <£18,000 | 1.38 | [1.25,1.53] | 5.70e-10 | 1.35 | [1.14,1.59] | 5.32e-04 | 1.41 | [1.24,1.6] | 2.39e-07 |
| £18,000-£30,000 | 1.16 | [1.07,1.26] | 3.69e-04 | 1.14 | [1,1.3] | 5.52e-02 | 1.18 | [1.06,1.31] | 2.06e-03 |
| £52,000-£100,000 | 0.90 | [0.83,0.97] | 4.32e-03 | 0.88 | [0.78,0.99] | 3.03e-02 | 0.90 | [0.82,1] | 4.93e-02 |
| >£100,000 | 0.70 | [0.63,0.79] | 1.80e-09 | 0.66 | [0.56,0.78] | 1.73e-06 | 0.73 | [0.63,0.86] | 9.95e-05 |
| **Educational attainment** (ref: University degree) |  |  |  |  |  |  |  |  |  |
| Secondary | 0.96 | [0.9,1.04] | 3.27e-01 | 0.87 | [0.77,0.98] | 1.87e-02 | 1.05 | [0.95,1.15] | 3.41e-01 |
| Further | 1.03 | [0.95,1.13] | 4.52e-01 | 1.05 | [0.92,1.21] | 4.64e-01 | 1.03 | [0.92,1.15] | 5.75e-01 |
| Vocational | 0.92 | [0.83,1.02] | 1.13e-01 | 0.77 | [0.67,0.9] | 5.78e-04 | 1.07 | [0.93,1.23] | 3.30e-01 |
| **Neighbourhood deprivation** (per SD) | 1.01 | [1,1.02] | 9.01e-02 | 1.01 | [1,1.03] | 1.54e-01 | 1.01 | [0.99,1.02] | 2.46e-01 |
| **Social isolation** (ref: No) |  |  |  |  |  |  |  |  |  |
| Yes | 0.91 | [0.83,1.01] | 6.49e-02 | 0.94 | [0.81,1.09] | 4.12e-01 | 0.89 | [0.78,1.01] | 7.60e-02 |
| Missing data | 0.78 | [0.48,1.28] | 3.26e-01 | 0.80 | [0.38,1.68] | 5.46e-01 | 0.84 | [0.43,1.61] | 5.89e-01 |
| **Self-help** (ref: No) |  |  |  |  |  |  |  |  |  |
| Yes | 1.98 | [1.83,2.14] | 1.00e-66 | 2.51 | [2.19,2.89] | 4.79e-38 | 1.76 | [1.6,1.93] | 3.60e-32 |
| **Self-medication with alcohol or drugs** (ref: No) |  |  |  |  |  |  |  |  |  |
| Yes | 0.95 | [0.88,1.02] | 1.82e-01 | 0.85 | [0.76,0.94] | 2.21e-03 | 1.07 | [0.96,1.2] | 2.10e-01 |

| **Treatment receipt** | **Full sample** | | | **Male sample** | | | **Female sample** | | |
| --- | --- | --- | --- | --- | --- | --- | --- | --- | --- |
| Variable | OR | 95% CI | P | OR | 95% CI | P | OR | 95% CI | P |
| **Age** (per year) | 1.00 | [0.99,1] | 3.84e-01 | 1.00 | [0.99,1] | 0.284000 | 1.00 | [0.99,1] | 7.90e-01 |
| **Sex** (ref: Female) |  |  |  |  |  |  |  |  |  |
| Male | 0.84 | [0.77,0.9] | 4.21e-06 | NA | NA | NA | NA | NA | NA |
| **UK ethnic minority** (ref: No) |  |  |  |  |  |  |  |  |  |
| Yes | 0.59 | [0.48,0.72] | 3.80e-07 | 0.56 | [0.39,0.81] | 0.001860 | 0.60 | [0.47,0.77] | 6.00e-05 |
| Missing data | 0.78 | [0.4,1.51] | 4.55e-01 | 0.86 | [0.33,2.2] | 0.744000 | 0.70 | [0.28,1.8] | 4.64e-01 |
| **Household income** (ref: £30,000-£52,000) |  |  |  |  |  |  |  |  |  |
| <£18,000 | 1.35 | [1.19,1.53] | 1.79e-06 | 1.45 | [1.17,1.81] | 0.000910 | 1.30 | [1.12,1.5] | 6.58e-04 |
| £18,000-£30,000 | 1.19 | [1.07,1.31] | 8.67e-04 | 1.26 | [1.06,1.51] | 0.007990 | 1.15 | [1.01,1.29] | 3.13e-02 |
| £52,000-£100,000 | 0.95 | [0.86,1.04] | 2.57e-01 | 1.05 | [0.9,1.23] | 0.528000 | 0.89 | [0.79,1] | 5.43e-02 |
| >£100,000 | 1.12 | [0.95,1.31] | 1.86e-01 | 1.20 | [0.92,1.55] | 0.175000 | 1.07 | [0.87,1.32] | 5.24e-01 |
| **Educational attainment** (ref: University degree) |  |  |  |  |  |  |  |  |  |
| Secondary | 0.85 | [0.78,0.93] | 5.12e-04 | 0.86 | [0.74,1.01] | 0.061100 | 0.85 | [0.76,0.95] | 3.46e-03 |
| Further | 0.85 | [0.76,0.94] | 1.92e-03 | 0.95 | [0.79,1.15] | 0.625000 | 0.80 | [0.71,0.91] | 6.94e-04 |
| Vocational | 0.83 | [0.73,0.94] | 3.04e-03 | 0.81 | [0.66,0.98] | 0.032400 | 0.85 | [0.72,1] | 4.36e-02 |
| **Neighbourhood deprivation** (per SD) | 1.03 | [1.01,1.04] | 1.52e-04 | 1.02 | [0.99,1.04] | 0.202000 | 1.04 | [1.02,1.05] | 2.07e-04 |
| **Social isolation** (ref: No) |  |  |  |  |  |  |  |  |  |
| Yes | 1.12 | [0.99,1.28] | 7.71e-02 | 1.25 | [1.01,1.55] | 0.036300 | 1.05 | [0.89,1.23] | 5.73e-01 |
| Missing data | 1.54 | [0.71,3.35] | 2.71e-01 | 1.97 | [0.47,8.3] | 0.355000 | 1.38 | [0.55,3.47] | 4.89e-01 |
| **Self-help** (ref: No) |  |  |  |  |  |  |  |  |  |
| Yes | 1.28 | [1.17,1.39] | 1.92e-08 | 1.32 | [1.12,1.54] | 0.000685 | 1.26 | [1.14,1.4] | 6.38e-06 |
| **Self-medication with alcohol or drugs** (ref: No) |  |  |  |  |  |  |  |  |  |
| Yes | 1.09 | [0.99,1.2] | 7.34e-02 | 1.04 | [0.9,1.21] | 0.613000 | 1.13 | [1,1.29] | 5.34e-02 |

### S.Table 5. Two-sample Z-tests

**S.Table 5. Differences in effect sizes between: univariable and multivariable models and: males and females. Tested using two-sample Z-tests**

**Treatment-seeking**

| Comparison | Variable | OR_1_ | | OR_2_ | | Z_diff_ | | P_diff_ |
| --- | --- | --- | --- | --- | --- | --- | --- | --- |
| *Full sample (Univariable versus Multivariable effects)* | Age | 0.99 | | 1.00 | | -3.89 | | 0.00010 |
|  | Self-medication (alc/drugs) | 1.12 | | 0.95 | | 3.29 | | 0.00100 |
|  | Self-help | 2.30 | | 1.98 | | 2.88 | | 0.00398 |
| *Male sample (Univariable versus Multivariable effects)* | Self-medication (alc/drugs) | 1.1 | | 0.85 | | 3.60 | | 0.000318 |
|  | Age | 1.0 | | 1.01 | | -2.12 | | 0.034000 |
| *Female sample (Univariable versus Multivariable effects)* | Self-medication (alc/drugs) | 1.32 | | 1.07 | | 2.79 | | 0.00527 |
|  | Age | 0.99 | | 0.99 | | -2.50 | | 0.01240 |
|  | Self-help | 2.01 | | 1.76 | | 2.15 | | 0.03160 |
| *Univariable effects (Female versus Male sample)* | Age | 0.99 | | 1.00 | | -5.27 | | 1.00e-07 |
|  | Self-help | 2.01 | | 2.63 | | -3.58 | | 3.44e-04 |
|  | Self-medication (alc/drugs) | 1.32 | | 1.10 | | 2.75 | | 5.96e-03 |
|  | Educational attainment (Voc.) | 1.08 | | 0.90 | | 2.05 | | 4.04e-02 |
| *Multivariable effects (Female versus Male sample)* | Age | | 0.99 | | 1.01 | | -4.48 | 7.50e-06 |
|  | Self-help | | 1.76 | | 2.51 | | -4.18 | 2.92e-05 |
|  | Educational attainment (Voc.) | | 1.07 | | 0.77 | | 3.16 | 1.58e-03 |
|  | Self-medication (alc/drugs) | | 1.07 | | 0.85 | | 3.05 | 2.29e-03 |
|  | Educational attainment (Sec.) | | 1.05 | | 0.87 | | 2.44 | 1.47e-02 |

**Treatment-receipt**

| Comparison | Variable | OR_1_ | OR_2_ | Z_diff_ | P_diff_ |
| --- | --- | --- | --- | --- | --- |
| *Full sample (Univariable versus Multivariable effects)* | Self-medication (alc/drugs) | 1.25 | 1.09 | 2.04 | 0.0414 |
| *Univariable effects (Female versus Male sample)* | Educational attainment (Further) | 0.82 | 1.02 | -2.05 | 0.0404 |

### S.Table 6. VIFS

**S.Table 6. Variance inflation factors**

| Variable | VIF |
| --- | --- |
| Household income<£18,000 | 1.24 |
| Household income£18,000-£30,000 | 1.24 |
| Household income£52,000-£100,000 | 1.24 |
| Household income>£100,000 | 1.24 |
| Age | 1.13 |
| Educational attainmentSecondary | 1.11 |
| Educational attainmentFurther | 1.11 |
| Educational attainmentVocational | 1.11 |
| Neighbourhood deprivation | 1.08 |
| Self medication with alcohol or drugs | 1.05 |
| Sex | 1.04 |
| Social isolation | 1.04 |
| Self help | 1.03 |
| UK ethnic minority | 1.02 |
| Lifetime symptom severity | 1.02 |

### S.Table 7. Interaction effects

**S.Table 7. Summary statistics from multivariable regression analyses testing for associations between treatment-seeking and sociodemographic variables with and without inclusion of selected interaction terms**

|  | **Main effects model** | | | **Interaction effects model** | | |
| --- | --- | --- | --- | --- | --- | --- |
| Variable | OR | 95% CI | P | OR | 95% CI | P |
| **Age** (per year) | 1.00 | [1,1.01] | 1.93e-01 | 0.99 | [0.99,1] | 3.17e-02 |
| **Sex** (ref: Female) |  |  |  |  |  |  |
| Male | 0.63 | [0.6,0.67] | 6.80e-52 | 0.20 | [0.12,0.33] | 2.87e-10 |
| **UK ethnic minority** (ref: No) |  |  |  |  |  |  |
| Yes | 0.72 | [0.6,0.85] | 1.37e-04 | 0.72 | [0.6,0.85] | 1.37e-04 |
| Missing ethnicity data | 0.63 | [0.39,1.04] | 6.98e-02 | 0.63 | [0.38,1.03] | 6.73e-02 |
| **Household income** (ref: £30,000-£52,000) |  |  |  |  |  |  |
| <£18,000 | 1.38 | [1.25,1.53] | 5.70e-10 | 1.38 | [1.25,1.54] | 5.75e-10 |
| £18,000-£30,000 | 1.16 | [1.07,1.26] | 3.69e-04 | 1.16 | [1.07,1.26] | 3.25e-04 |
| £52,000-£100,000 | 0.90 | [0.83,0.97] | 4.32e-03 | 0.89 | [0.83,0.96] | 4.06e-03 |
| >£100,000 | 0.70 | [0.63,0.79] | 1.80e-09 | 0.70 | [0.62,0.78] | 1.01e-09 |
| **Educational attainment** (ref: University degree) |  |  |  |  |  |  |
| Secondary | 0.96 | [0.9,1.04] | 3.27e-01 | 1.03 | [0.92,1.15] | 6.13e-01 |
| Further | 1.03 | [0.95,1.13] | 4.52e-01 | 1.04 | [0.95,1.15] | 3.68e-01 |
| Vocational | 0.92 | [0.83,1.02] | 1.13e-01 | 1.07 | [0.93,1.23] | 3.39e-01 |
| **Neighbourhood deprivation** (per SD) | 1.01 | [1,1.02] | 9.01e-02 | 1.01 | [1,1.02] | 6.91e-02 |
| **Social isolation** (ref: No) |  |  |  |  |  |  |
| Yes | 0.91 | [0.83,1.01] | 6.49e-02 | 0.91 | [0.83,1.01] | 6.50e-02 |
| Missing data | 0.78 | [0.48,1.28] | 3.26e-01 | 0.82 | [0.5,1.34] | 4.24e-01 |
| **Self-help** |  |  |  |  |  |  |
| Yes | 1.98 | [1.83,2.14] | 1.09e-66 | 1.75 | [1.6,1.93] | 5.07e-32 |
| **Self-medication with alcohol or drugs** |  |  |  |  |  |  |
| Yes | 0.95 | [0.88,1.02] | 1.82e-01 | 1.07 | [0.96,1.19] | 2.40e-01 |
| **Lifetime symptom severity** (per SD) | 2.29 | [2.2,2.38] | 0.00e+00 | 2.30 | [2.21,2.39] | 0.00e+00 |
| **Interaction effects** |  |  |  |  |  |  |
| Sex * Age |  |  |  | 1.02 | [1.01,1.03] | 1.28e-06 |
| Sex * Secondary education |  |  |  | 1.03 | [0.86,1.23] | 7.26e-01 |
| Sex * Further education |  |  |  | 0.84 | [0.72,0.97] | 1.81e-02 |
| Sex * Vocational education |  |  |  | 0.73 | [0.6,0.89] | 1.65e-03 |
| Sex * Self-help |  |  |  | 1.43 | [1.21,1.69] | 2.88e-05 |
| Sex * Self-medication |  |  |  | 0.80 | [0.69,0.93] | 3.73e-03 |

## S.Figures

### S.Fig 2. The gaps

**S.Fig 2. The treatment gap**

“Treatment gap = Seeking gap + Access gap”


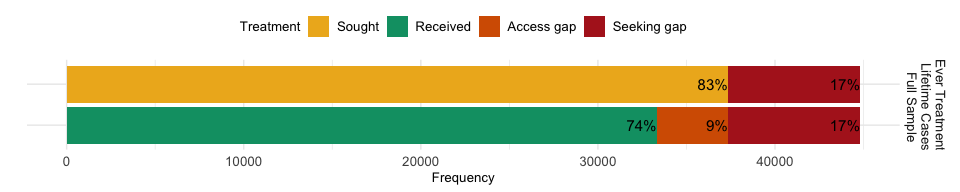


### S.Fig 3. Odds ratios

S.Fig 4. Factors associated with treatment-seeking and treatment-receipt **(ever)** in UK Biobank participants meeting CIDI-SF criteria for a **lifetime** diagnosis of generalised anxiety or major depressive disorder.


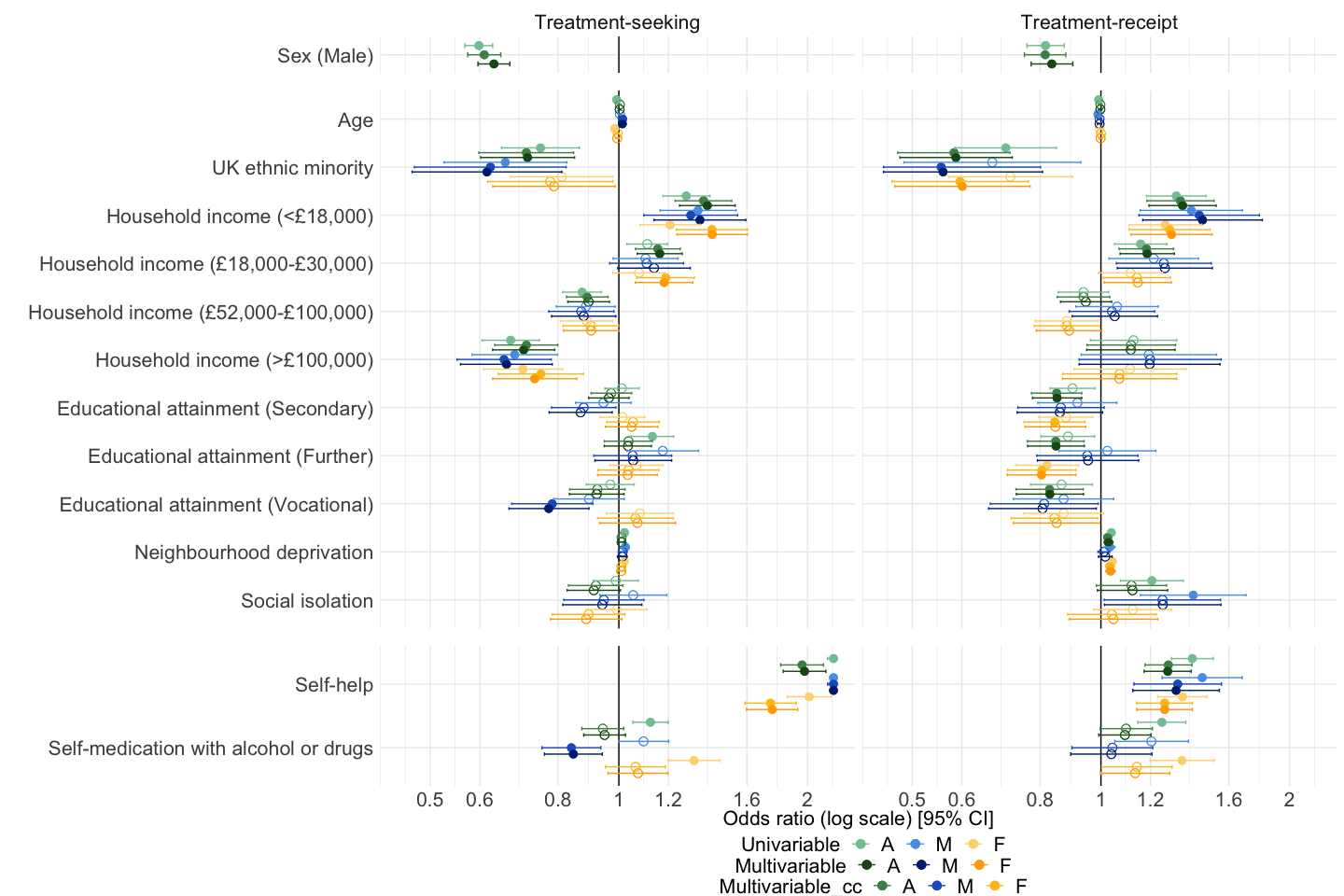


*Odds of treatment-seeking and treatment-receipt estimated in:* ***all (green)*** *UK Biobank participants meeting CIDI-SF criteria;* ***male (blue)*** *participants meeting CIDI-SF criteria;* ***female (yellow)*** *participants meeting CIDI-SF criteria. Estimated from three models: the primary* ***multivariable*** *model (adjusted for missing data;* ***darker shade****);* ***univariable*** *models (unadjusted effects;* ***lighter shade****);* ***multivariable*** *complete-case model (adjusted effect sizes estimated in participants with complete data across all variables;* ***mid shade****). Statistical significance threshold p < 0.0033, controlling for 15 effectively independent tests.*
